# Accurate models and nutritional strategies for specific oxidative stress factors: Does the dose matter in swine production?

**DOI:** 10.1186/s40104-023-00964-8

**Published:** 2024-01-26

**Authors:** Changming Hong, Yujian Huang, Shuting Cao, Li Wang, Xuefen Yang, Shenglan Hu, Kaiguo Gao, Zongyong Jiang, Hao Xiao

**Affiliations:** 1 State Key Laboratory of Swine and Poultry Breeding Industry, Ministry of Agriculture Key Laboratory of Animal Nutrition and Feed Science in South China, Guangdong Public Laboratory of Animal Breeding and Nutrition, Guangdong Provincial Key Laboratory of Animal Breeding and Nutrition, Maoming Branch, Guangdong Laboratory for Lingnan Modern Agriculture, Guangzhou, China; 2 Institute of Animal Science, Guangdong Academy of Agricultural Sciences, 1 Dafeng 1st Street, Guangzhou, 510640 China

**Keywords:** Accurate models, Dose, Nutritional strategies, Oxidative stress, Swine

## Abstract

Oxidative stress has been associated with a number of physiological problems in swine, including reduced production efficiency. Recently, although there has been increased research into regulatory mechanisms and antioxidant strategies in relation to oxidative stress-induced pig production, it remains so far largely unsuccessful to develop accurate models and nutritional strategies for specific oxidative stress factors. Here, we discuss the dose and dose intensity of the causes of oxidative stress involving physiological, environmental and dietary factors, recent research models and the antioxidant strategies to provide theoretical guidance for future oxidative stress research in swine.

## Introduction

Oxidative stress has been interpreted as an imbalance between oxidation and anti-oxidation (more oxidizing), which was first defined in 1985 [1]. Oxidative stress in pigs is often associated with other pathological factors, including metabolic disorders and placental dysfunction in sows [2], and poor growth performance in piglets [2, 3]. These factors have a direct impact on sow reproductive performance and piglet growth. Therefore, it is crucial to address oxidative stress in pig production. Current research efforts are primarily focused on alleviating oxidative stress in pigs by supplementing diets with antioxidants and free radical scavengers. However, the development of accurate models and nutritional strategies for specific oxidative stress factors has been largely unsuccessful.

The basis for the definition of oxidative stress is reactive oxygen species (ROS). ROS were the unregulated by-products of aerobic metabolism and other enzymatic processes that play a critical role in regulating cell function and biological processes. Uncontrolled production of ROS can overwhelm the ability of enzymatic and non-enzymatic antioxidant defence mechanisms, leading to a state of oxidative stress and consequently damage many biological macromolecules such as lipids, DNA and proteins [1]. The oxidative stress state can be measured, but find the specificity, has a strong correlation with physiological and pathological status of pigs accurately respond stress markers for oxidative stress measurement, specific display certain conditions, has prognostic value and structure stability of cost-effective, is particularly important to be used massively in pig production. In addition, dose ranges for indicators of whether pigs are under oxidative stress at different stages are also not yet well understood.

Here, we discuss the dose and dose intensity of the causes of oxidative stress including physiological status (pregnancy, lactation, neonatal or weaning stress), environmental factors (heat and cold stress) and dietary factors (dietary mycotoxins and lipid peroxidation) and regulatory metabolism in swine, the research models using different chemical compounds and also take into account the antioxidant strategies of nutritional regulation to provide theoretical guidance for the subsequent oxidative stress research.

## Causes of oxidative stress in swine

Oxidative stress in swine can be caused by various factors, including physiological factors, environmental factors, and dietary factors. These causes have been the focus of recent studies on stress responses and regulatory metabolism in swine [4]. In this section, we focus on the levels of different stress indexes and effects at different physiological stages and aim to provide a comprehensive understanding of the occurrence and mechanisms of oxidative stress in swine.

### Physiologically induced oxidative stress

#### Oxidative stress in sows—gestation and lactation

**Fig. 1:**
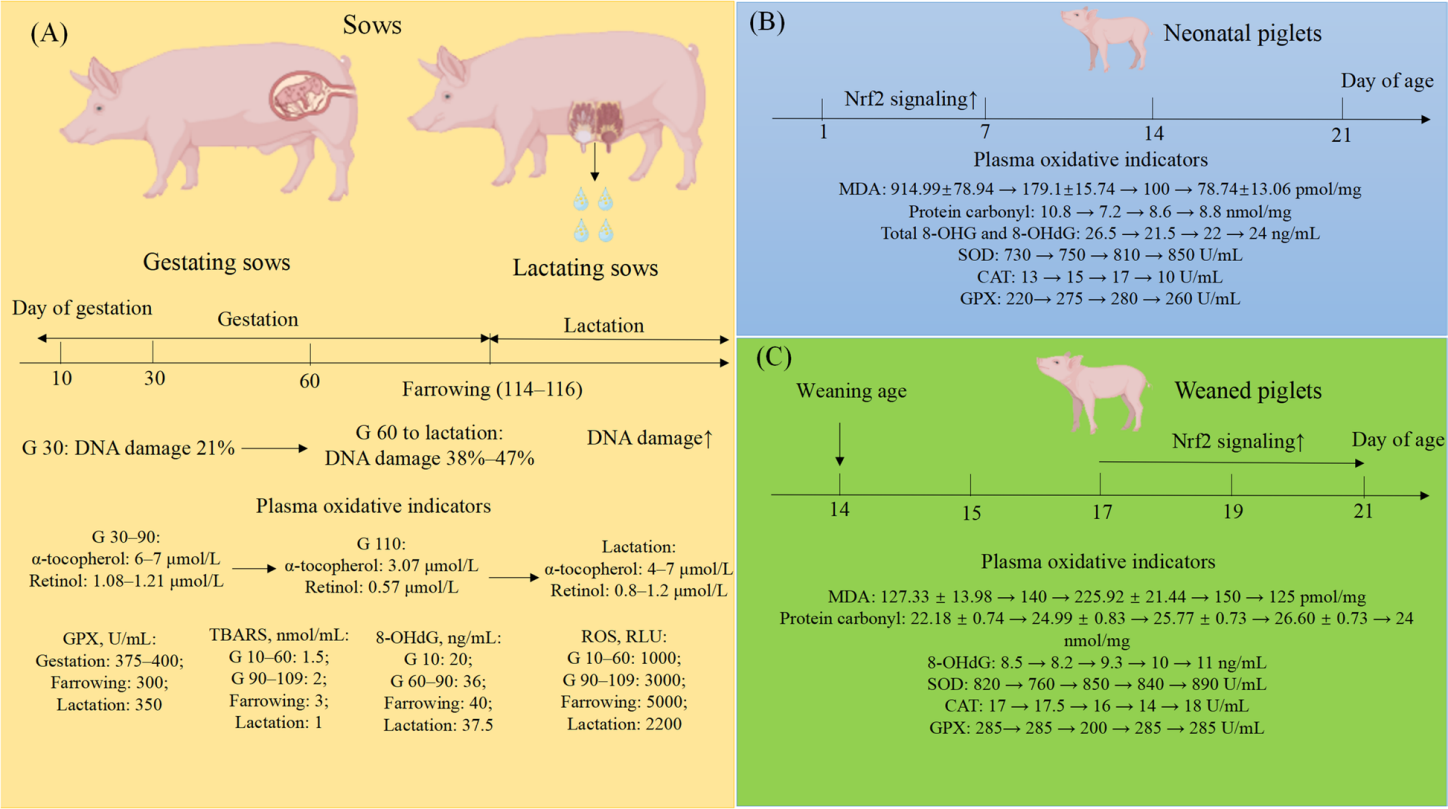
Overview of physiologically induced oxidative stress in swine. **A** The increased DNA damage and oxidative stress parameters were exhibited at the end of gestation and lactation in sows. **B** For neonatal piglets, the highest oxidative stress parameters levels were exhibited at 1 d after birth of neonatal piglets. After that the oxidative stress parameters were reduced by the activation of Nrf2 signaling, which were reduced at 7 d after birth. **C** For weaned piglets, the highest oxidative stress parameters levels were exhibited at 3 d after weaning of 14 d weaned piglets. After that, the oxidative stress parameters were reduced by the activation of Nrf2 signaling. SOD: superoxide dismutase; Gpx: glutathione peroxidase; CAT: catalase; T-AOC: total antioxidant capacity; MDA: malondialdehyde; H_2_O_2_: hydrogen peroxide; ROS: reactive oxygen species; 8-OHG: 8-hydroxyguanosine; 8-OHdG: 8-hydroxy-2 deoxyguanosine; TBARS: thiobarbituric acid reactive substances; RLU: relative light unit. The data in figure are come from the references [6–15]

It is well known that pregnant sows had increased oxidative stress during late gestation and lactation, which had an adverse effect on milk production, reproductive efficiency, and ultimately sow longevity [5]. Low DNA damage (21%) is present at d 30 of pregnancy (G 30), whereas increased DNA damage (38%–47%) is present throughout the gestational and lactational periods. Furthermore, plasma retinol and α-tocopherol concentration were reduced at the end of gestation (G 110) compared with G 30 [6]. Moreover, there is an increased systemic oxidative stress during late gestation and early lactation of sows, according to increasing levels of oxidative stress parameters, such as thiobarbituric acid reactive substances (TBARS), 8-hydroxy-2 deoxyguanosine (8-OHdG), and ROS [7] (Data are shown in Fig. 1). However, there is still lack studies focus on the reference value of oxidative stress in sows cause by gestation and lactation.

#### Oxidative stress in piglets—birth and weaning stress

Due to their underdeveloped antioxidant systems, neonatal piglets are unable to efficiently scavenge excessive free radicals, resulting in oxidative stress [8]. Oxidative stress parameters like plasma malondialdehyde (MDA), protein carbonyl, and total 8-hydroxyguanosine (8-OHG) and 8-OHdG levels were exhibited at very high level at 1 d, while gradually decreased with increasing age. Furthermore, compared with the 1 d, superoxide dismutase (SOD) and glutathione peroxidase (GPx) activities were significantly increased (*P* < 0.05) at d 7, 14, and 21 [9] (Data are shown in Fig. 1). In addition, piglets with intrauterine growth restriction (IUGR) exhibit oxidative stress characterized by lower antioxidant enzyme activities and increased lipid peroxidation in the liver compared to normal-birth piglets [10].

The effects of weaning stress on swine production have been well charactered [11–14], so this review will not describe them excessively. Generally, weaning stress could cause the changes in oxidative indictors that lead to oxidative stress in piglets (Fig. 1). For example, weaning induced oxidative stress was deteriorated until d 3 of weaning in 14 d weaned piglets. After that, the activation of nuclear factor erythroid 2-related factor 2 (Nrf2) signaling may contribute to the alleviation of weaning stress [15]. Additionally, mitophagy, a process of selective degradation of damaged mitochondria, has been regarded to play a role in the feedback alleviation of oxidative stress after weaning [16]. Therefore, these pathways may be efficient targets for the nutritional regulation to alleviate weaning stress in piglets. Unfortunately, these articles mainly determine whether oxidative stress occurs according to the control group, as there is little research on the reference value of oxidative stress in weaning stress based on age and weight.

### Environmentally induced oxidative stress

**Fig. 2:**
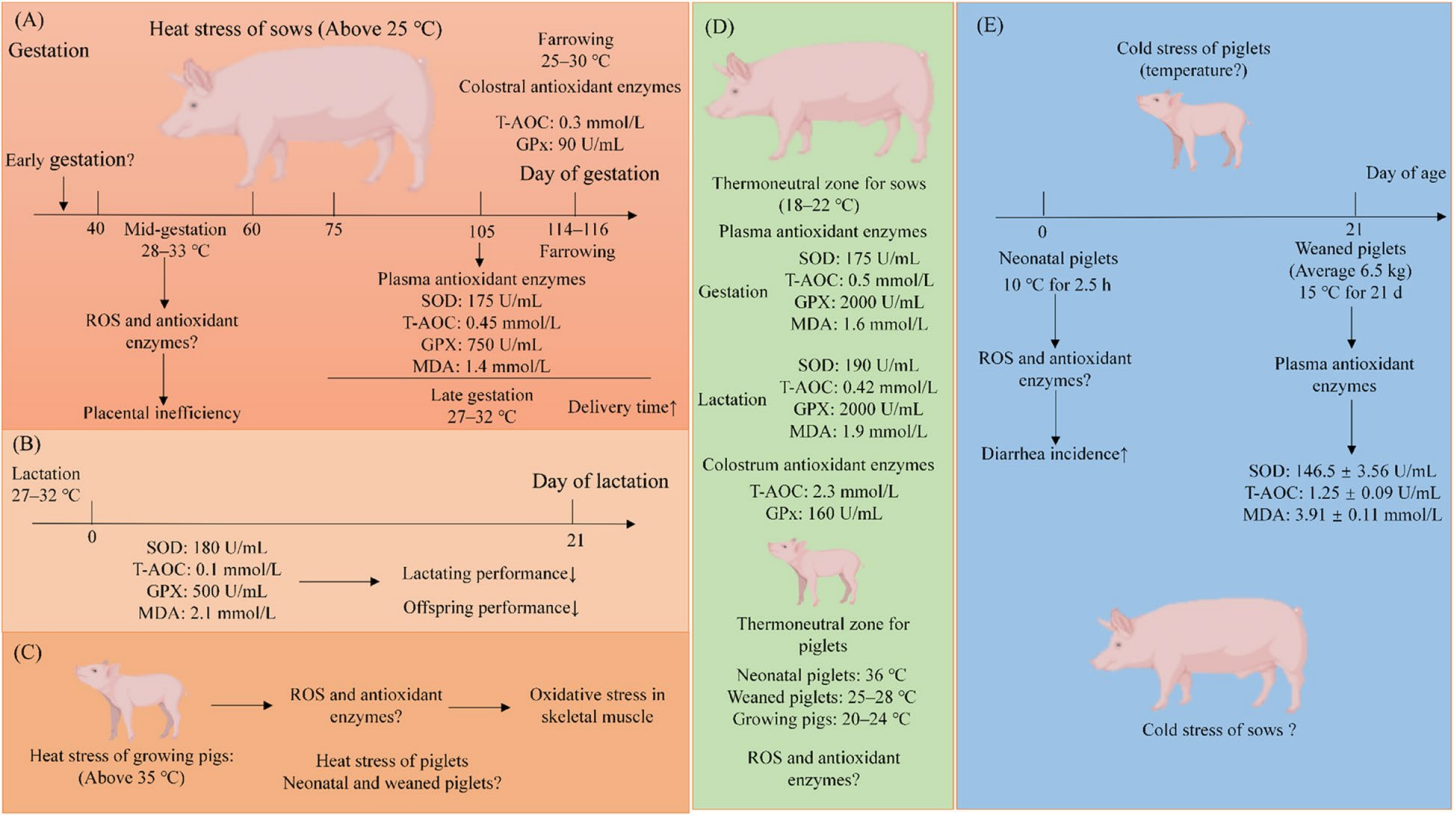
Overview of environmentally induced oxidative stress in swine. **A** At mid gestation, heat stress causes the placental inefficiency of sows. At late gestation and farrowing, heat stress causes the decrease of antioxidant capacity, which prolong the delivery time of sows. **B** At lactation, the decrease of antioxidant capacity causes the decreased lactating performance of sows and further reducing the offspring performance. **C** Heat stress could lead to oxidative stress in skeletal muscle of growing pigs. **D** The thermoneutral zone and levels of oxidative indictors for pigs at different stage. **E** Cold stress could cause the increased diarrhea incidence of neonatal piglets. SOD: superoxide dismutase; Gpx: glutathione peroxidase; CAT: catalase; T-AOC: total antioxidant capacity; MDA: malondialdehyde; H_2_O_2_: hydrogen peroxide; ROS: reactive oxygen species. The data in figure are come from the references [20–31]

Pigs in particular are highly sensitive to changes in ambient temperature [17–19]. Therefore, this part focuses on how different environmental temperature-induced stresses of pigs, such as heat stress and cold stress, affect the performance of pigs at different stages. We also provide the thermoneutral zone for pigs at different stages with the aim of providing better regulatory strategies to alleviate environmentally induced oxidative stress in pigs (Fig. 2).

#### Heat stress

Sows that are metabolically stressed during the perinatal period are more susceptible to high temperatures, resulting in heat stress and reduced reproductive function [18]. The most suitable temperature for sows is 18–22 °C according to a previous study [20]. During mid-gestation (G 40–60), high summer temperatures (33 °C between 0900 and 1700 h, and 28 °C between 1700 and 0900 h) could lead to placental inefficiency in sows compared to a thermoneutral environment (constant 20 °C) [21]. During late gestation, compared to spring temperatures (from March to May, approximately 15–24 °C), high summer temperatures (from July to September, approximately 27.5–30 °C) reduced the antioxidant activities of plasma at G 105 (Gpx decreased from 2,000 to 750 U/mL) and colostrum at farrowing (Total antioxidant capacity (T-AOC) decreased from 2.3 to 0.3 mmol/L and Gpx decreased from 160 to 90 U/mL) in sows [22], which may prolong the parturition period of sows and result in poor growth performance of the offspring piglets [20, 23]. Around farrowing, sows exposed to high ambient temperatures (around 25–30 °C) also have an unfavourable effect on sow reproductive performance [24, 25]. Furthermore, at high summer temperatures (27–30 °C), lactating sows showed lower plasma antioxidant capacity (T-AOC decreased from 0.45 to 0.1 mmol/L and Gpx decreased from 2,000 to 500 U/mL) compared to those exposed to a thermoneutral environment (15–24 °C), which led to a decrease in lactation performance of sows, resulting in reduced performance of their offspring [22, 26]. For growing pigs, previous studies described that heat stress (35 °C for 1 or 3 d) could induce oxidative stress in skeletal muscle of growing pigs (35 ± 4 kg), possibly by inhibiting mitophagy and accumulation of damaged mitochondria in muscle cells [27, 28]. In addition, short-term heat stress (37 °C for 2–6 h) could induce oxidative stress in the muscle of growing pigs (63.8 ± 2.9 kg) [29]. These results suggest that heat stress has different effects at different stages in pigs. Unfortunately, there are few studies on the effects of heat stress on the performance of sows during early pregnancy and on neonatal and weaned piglets, and few studies focus on the value of oxidative stress in heat stress based on the stage of pigs, which needs further investigation.

#### Cold stress

Cold stress is another environmental challenge for swine, especially neonatal piglets. Previous research revealed that when exposed to a cold environment (10 °C) as opposed to a thermoneutral environment (36 °C), there was a decrease in the acquisition of colostral immunoglobulin. This resulted in an increase in the incidence of diarrhea and may have contributed to preweaning mortality in newborn piglets at 14.5 h of age [30]. Piglets exposed to a cold environment (15 °C) for 21 d showed higher antioxidant activities (T-AOC increased from 0.87 ± 0.05 to 1.25 ± 0.09 U/mL) than piglets exposed to a thermoneutral environment (26 °C), even though the animals' growth performance was diminished [31]. This may be because the piglets have become more adapted to prolonged cold exposure, as evidenced by the growth of oxidative muscle and gut bacteria [32–34]. Unfortunately, it is not yet known what temperature range causes cold stress in sows and weaned piglets. Furthermore, few studies have been conducted on the reference value of oxidative stress in piglets caused by cold stress. These should be prioritised in view of the rapid growth of the swine industry .

In conclusion, environmental challenges such as heat and cold stress may contribute significantly to oxidative stress in pig production. The possible thermoneutral zone and normal ranges of oxidative indicators for sows, neonatal and weaned piglets, and growing pigs are shown in Fig. 2.

### Dietary-induced oxidative stress                                                                                                   

Expect for physiologically and environmentally induced oxidative stress in swine, the dietary factors such as dietary mycotoxins pollution and lipid peroxidation could also cause oxidative stress in swine. In this section, the effects of different dose intensity of dietary mycotoxins and lipid peroxidation on the oxidative factors in various swine models will be summarized (Table 1).

**Table 1:** Dietary-induced oxidative stress in swine

Swine models	Body weight, kg	Inducer and duration	Changes in oxidative indicators	References
Weaned piglets	12.36 ± 0.26	DON 4 mg/kg, 30 d (From 28 to 57 days of age)	Serum: D 15: H_2_O_2_ (12 → 23 mmol/L) ↑ MDA (1 → 6.2 mmol/L) ↑ D 30: CAT (5.5 → 3.5 U/mL) ↓ H_2_O_2_ (12 → 22 mmol/L) ↑ MDA (2 → 3 nmol/mL) ↑	[35]
	6.97 ± 0.1	DON 3.8 mg/kg, 28 d (From 21 to 49 days of age)	Serum: GSH (4.24 → 2.8 mg/L) ↓ T-AOC (2.31 → 1.56 U/mL) ↓ MDA (1.96 → 3.31 mmol/L) ↑ Ileum (μmol/g protein): GSH (5.13 → 4.21) ↓, SOD (58.24 → 39.57) ↓ T-AOC (0.49 → 0.31) ↓, MDA (0.52 → 1) ↑ Jejunum (μmol/g protein): GSH (10.93 → 10.02) ↓, SOD (63.66 → 49.95) ↓ T-AOC (0.4 → 0.29) ↓, MDA (0.61 → 0.8) ↑	[36, 37]
	10.9 ± 0.77	OTA 0.25 mg/kg, 28 d	Liver: T-AOC (27 → 22 μmol/g tissue) ↓ TBARS (3.5 → 5.4 nmol/g tissue) ↑ Kidney: GPx (1.8 → 22 μmol/min/g tissue) ↓ T-AOC (23 → 19 μmol/g tissue) ↓ TBARS (0.8 → 1.2 nmol/g tissue) ↑	[38]
	6.97 ± 0.1	AFB1 0.32 mg/kg, 30 d (From 28 to 57 days of age)	MLNs: SOD (400 → 352 U/g tissue) ↓ CAT (4 → 2.92 μmol/min/g tissue) ↓ GPx (4.8 → 3.74 μmol/min/g tissue) ↓ T-AOC (11 → 8.75 μmol/g tissue) ↓	[39]
Growing pigs	16.3 ± 1.5	DON 3–12 mg/kg, 21 d (From 67 to 88 days of age)	Serum (U/mL): 3 mg/kg: SOD (117.5 ± 6.26 → 100.88 ± 14.24) ↓ 6 mg/kg: SOD (117.5 ± 6.26 → 84.48 ± 8.97) ↓ GPx (3,424.7 ± 147.5 → 2,967.3 ± 124.4) ↓ 12 mg/kg: SOD (117.5 ± 6.26 → 79.94 ± 15.62) ↓ GPx (3,424.7 ± 147.5 → 2,521.3 ± 334.6) ↓	[40, 41]
Nursery pigs	Average 6.5	Oxidized soybean oil 60 g/kg, 35 d (From 21 to 66 days of age)	Jejunal mucosa: MDA (35 → 54 μmol/g protein) ↑	[42]
Newborn piglets	1.8 ± 0.04	Oxidized soybean oil 50 g/kg, 21 d (From 4 to 25 days of age)	Jejunal mucosa: MDA (3.17 → 5.2 mol/L) ↑ GSSG (8.58 → 12.62 mmol/g protein) ↑	[43]

*CAT* Catalase, *GPx* Glutathione peroxidase, *SOD* Superoxide dismutase, *MLNs* Mesenteric lymph nodes, *DON* Deoxynivalenol, *OTA* Ochratoxin A, *AFB1* Aflatoxin B1, *ZEA* Zearalenone

#### Dietary mycotoxins-induced oxidative stress

The contamination of feed ingredients and complete feeds with these mycotoxins has been a major concern in China, especially in recent years [44]. Numerous studies have shown that the presence of these feed mycotoxins can lead to oxidative stress in pigs, reducing growth performance and meat quality [45–48]. In particular, the mycotoxin deoxynivalenol (DON) can induce oxidative stress and inflammation in the pig intestine, which has been extensively studied as a model of oxidative stress. Research has shown that supplementation of the basal diet of 28-day-old weaned piglets with 4 mg/kg DON resulted in a reduction in blood catalase (CAT) concentration [35]. Moreover, our recent studies have shown that dietary supplementation with 3.8 mg/kg DON reduced the intestinal antioxidant capacity and induced intestinal inflammation in 21 d weaned piglets [36, 37]. Dietary supplementation with 3–12 mg/kg DON could cause a decrease in antioxidant capacity in a dose-dependent manner, leading to oxidative stress in growing pigs [40, 41]. These results indicate that the peroxidative effects of DON on pigs are dependent on the dose of DON, the stage and the body weight of the pigs. In addition, exposure of piglets to 0.25 mg/kg ochratoxin A (OTA) reduced antioxidant capacity in the liver and kidney [38]. Moreover, dietary supplementation with 0.32 mg/kg aflatoxin B1 (AFB1) decreased the antioxidant status in piglet mesenteric lymph nodes (MLNs) [39], which may contribute to intestinal barrier dysfunction [49]. However, future studies should focus on oxidative stress caused by dietary mycotoxins based on dosage and stage, age or weight of the pigs.

#### Dietary lipid peroxidative induced stress

Lipids are susceptible to be oxidized at high temperatures and produce large amounts of lipid peroxides (e.g., MDA and 4-hydroxynonenal (4-HNE)), which could lead to lipid peroxidation in animals [50, 51]. Therefore, proper assessment of lipid peroxidation has been an important part of quality control for the prevention of oxidative stress and performance loss in pigs [52]. Dietary supplementation with 60 g/kg oxidized soybean oil (MDA level = 4.5 mmol/L oil) increased lipid peroxidation (characterized by MDA levels ranging from 35 to 54 μmol/g protein in the jejunal mucosa) of nursery pigs [42]. Our previous studies demonstrated that dietary supplementation with 50 g/kg oxidized fish oil (peroxide level = 186.89 mmol/kg) significantly increased the levels of MDA and glutathione oxidized (GSSG) in the jejunal mucosa of newborn piglets compared to those dietary added 50 g/kg fresh fish oil (peroxide level = 4.20 mmol/kg) [43]. Cellular models of 4-HNE-induced oxidative stress have also been used to study the peroxidative effects of a lipid peroxidation diet on pigs [53, 54]. These studies found that 4-HNE could induce an increase in cellular ROS generation and DNA damage in IPEC-1 cells [55, 56]. However, lipid peroxidation in swine diets has not yet been well studied.

### Chemical compounds-induced oxidative stress models in swine

Many chemical compounds (e.g., diquat, hydrogen peroxide (H_2_O_2_) and lipopolysaccharide (LPS)) have also been used to investigate the underlying mechanism of oxidative stress in swine. Here, we will discuss the regulatory mechanisms of each of the oxidative stress models, and then compare their respective characteristics and assess their impact on the pig mode (Table 2).

**Table 2:** Chemical compounds-induced oxidative stress models in swine

Swine models	Body weight, kg	Inducer and duration	Changes in oxidative indicators	References
Weaned piglets	9.92 ± 0.3	Diquat 10 mg/kg, 7 d	Serum: T-AOC (2.5 → 2.1 U/L) ↓ MDA (2.8 → 4.5 nmol/L) ↑	[57]
	10.63 ± 1	Diquat 10 mg/kg, 7 d (From 35 to 42 days of age)	Serum: SOD (74.8 ± 1.5 → 56.14 ± 1.58 U/mL) ↓ CAT (10.35 ± 0.53 → 8.97 ± 0.34 U/mL) ↓ GPx (493.62 ± 30.95 → 313.56 ± 48.67 U/mL) ↓ MDA (2.76 ± 0.07 → 3.32 ± 0.11 mmol/L) ↑	[58]
	Average 9.6	Diquat 10 mg/kg, 7 d (From 35 to 42 days of age)	Jejunum: SOD (100.32 ± 11.29 → 60.06 ± 8.01 U/mg protein) ↓ GPx (99.35 ± 8.31 → 57.88 ± 6.4 U/mg protein) ↓ MDA (0.54 ± 0.07 → 1.91 ± 0.1 nmol/g protein) ↑ Mitochondrial ROS (9 times higher than control) ↑	[59]
	10.96 ± 0.61	H_2_O_2_ 1 mg/kg, 7 d	Serum: MDA (3.8 → 4.5 ng/mL) ↑ H_2_O_2_ (20 → 28 mmol/L) ↓	[60]
	11.58 ± 0.26	LPS 100 μg/kg, 3 h (On 35 days of age)	Serum: SOD (110.6 ± 6.9 → 98.3 ± 5.8 U/mL) ↓ CAT (4.75 ± 0.51 → 4.1 ± 0.36 U/mL) ↓ GPx (396.6 ± 18.5 → 359.4 ± 36.8 U/mL) ↓ H_2_O_2_ (51.28 ± 8.09 → 65.05 ± 8.46 mol/L) ↑ MDA (5.63 ± 0.46 → 6.59 ± 1 mmol/L) ↑	[61]
	Average 9.8	LPS 100 μg/kg, 7 d (From 35 to 42 days of age)	Jejunum: SOD (95.85 ± 3.13 → 63.12 ± 2.97 U/mg protein) ↓ GPx (72.11 ± 3.51 → 53.99 ± 3.26 U/mg protein) ↓ MDA (0.95 ± 0.16 → 1.51 ± 0.25 nmol/g protein) ↑ Mitochondrial ROS (8 times higher than control) ↑	[62]
	6.65 ± 1.19	LPS 100 μg/kg, 5 d (From 31 to 36 days of age)	Serum: T-AOC (0.21 ± 0.008 → 0.16 ± 0.025 mmol/L) ↓ SOD (27.73 ± 1.19 → 24.73 ± 1.59 U/mL) ↓ GPx (317.2 ± 28.96 → 245.63 ± 21.37 U/mL) ↓	[63]
100-day-old male miniature pigs	21.73 ± 0.43	CTX 50 mg/kg, 7 d	Serum: SOD (27 → 8 U/L) ↓ GPx (420 → 220 μmol/L) ↓ MDA (12 → 1.51 ± 24 μmol/L) ↑	[64]

*BW* Body weight, *CAT* Catalase, *SOD* Superoxide dismutase, *GPx* Glutathione peroxidase, *MDA* Malondialdehyde, *ROS* Reactive oxygen species, *H*_*2*_*O*_*2*_ Hydrogen peroxide, *GSH* Glutathione, *T-AOC* Total antioxidant capacity, *LPS* Lipopolysaccharide

#### Diquat-induced oxidative stress models

Diquat is a herbicide that is commonly used to induce oxidative stress in animal models due to its duration [65], making it a suitable model to study how oxidative stress affects physiological changes in swine [66]. Injection of 10 mg/kg diquat for 7 d reduced T-AOC levels and increased MDA levels, causing intestinal damage and reducing growth performance in weaned piglets [57]. Furthermore, 35-day-old weaned piglets treated with 10 mg/kg diquat for 7 d showed a significant decrease of antioxidant capacity and increased MDA levels [58], which could lead to mitochondrial dysfunction and further impairment of intestinal barrier function [59]. Eventually, diquat leads to an excess of oxygen radicals and aminotransferase in pigs, which disturbs the redox balance in the liver or gut. In conclusion, models of diquat-induced oxidative stress are focused on weaned piglets between 9–10 kg, and the dose of diquat is 10 mg/kg (Table 2).

#### H_2_O_2_-induced oxidative stress models

H_2_O_2_-induced oxidative stress in porcine intestine epithelium cells, as shown by elevated ROS levels, resulting in reduced cell viability [67]. Intragastric or intraperitoneal treatment with 100 μmol/L H_2_O_2_ caused oxidative stress in intestinal epithelial cells through the blockade of T-AOC [68]. 1 mg/kg H_2_O_2_ increased serum MDA and H_2_O_2_ levels in piglets by activation of the nuclear factor-κB (NF-κB) and Nrf2 signaling pathways and induction of the autophagic response in the jejunum [60, 69]. Furthermore, high doses H_2_O_2_ (10 mg/kg for piglet model (11.29 ± 0.32 kg) or 100 μmol/L for cell model) promoted mitochondrial dysfunction, whereas low doses H_2_O_2_ (5 mg/kg for piglet model or 50 μmol/L for cell model) showed a feedback regulatory mechanism against mitochondrial oxidative damage by increasing uncoupling protein 2 (UCP2) expression and mitochondrial proton leak [70]. Mitophagy may be involved in the above feedback control to improve oxidative stress (induced by 600 μmol/L H_2_O_2_)-induced intestinal barrier degradation [71]. In conclusion, H_2_O_2_ is the direct inducer of cellular oxidative stress. However, the H_2_O_2_-induced oxidative stress model has significant limitations in its application to pigs because pigs must first undergo surgery, which can easily lead to gastrointestinal ulceration in pigs, reducing their survival rates.

#### LPS-induced oxidative stress models

LPS, a lipid and polysaccharide endotoxin, has been used to induce an inflammatory response in most animal studies [72]. However, because of the link between the inflammatory response and oxidative stress [73], the LPS model has also been used as a model of oxidative stress in porcine research. For example, LPS at 100 mg/kg (intraperitoneal injection) caused intestinal oxidative stress in weaned piglets, as indicated by decreased jejunal T-AOC and GSH levels and increased MDA levels [74]. Piglets exposed to LPS also showed impaired intestinal barrier and mitochondrial function, as well as facilitated intestinal mitophagy [62]. In addition, after continuous low-dose induction of LPS, weaned piglets may have developed tolerance to endotoxin. LPS-induced oxidative stress was observed at an early stage (within 5 d of LPS stimulation), but was attenuated at a later stage (after 5 days of LPS stimulation) [63]. Overall, LPS induces oxidative stress in animals, mostly through the induction of inflammatory responses. Therefore, the LPS-induced oxidative stress paradigm is excellent for studying nutrients that have both antioxidant and anti-inflammatory properties, although individual studies of oxidative stress are less effective.

#### Other chemical compounds induced oxidative stress models

Other chemicals (including D-galactose and cyclophosphamide (CTX)) have been used in pigs. In IPEC-J2 cells, treatment with 500 µmol/L CTX resulted in a significant decrease in the activity of antioxidant enzymes and a significant increase in the MDA level [75]. Furthermore, dietary treatment with 50 mg/kg CTX dramatically decreased antioxidant enzyme activities while significantly increasing MDA levels [64]. Dietary D-galactose at 10 g/kg BW significantly increased blood MDA levels while decreasing intestinal antioxidant capacity in weaned piglets [76]. Although few studies have been conducted in porcine models, these findings suggest that CTX and D-galactose may be potential inducers of oxidative stress. In addition, the specific doses of the chemical compounds in pigs at different stages of oxidative stress should also be taken into consideration.

## Nutritional regulation strategies for oxidative stress in swine production

Recently a number of nutrients have been in use for the reduction of oxidative stress in pig production [77–79]. In this section, we mainly review current research on polyphenols and functional amino acids to reduce oxidative stress in pig production.

### Polyphenols

Polyphenols, the secondary plant metabolites with antioxidant properties, are attractive feed additives for nutritional management in pig production [80, 81]. In this section, we review current research on polyphenols for the reduction of oxidative stress in pigs (Table 3).

**Table 3:** The antioxidant effects of polyphenols on swine

Polyphenols	Dosages and duration	Swine models	Antioxidant effects	References
Daidzein	50 mg/kg 72 d	Weaned piglets 23-day-old 7.35 ± 0.14 kg BW	Plasma: D 14: MDA (2.52 → 2.31 mmol/L) ↓ D 42: SOD (18.19 → 20.54 U/mL) ↑	[82]
	640 mg/kg 64 d	Finishing pigs Average 57 kg BW	Liver (U/mg protein): CAT (79.5 → 96.3) ↑, SOD (285 → 358) ↑ longissimus muscle (U/mg protein): T-AOC (0.036 → 0.063) ↑, SOD (14.71 → 17.56) ↑	[83]
	40 mg/kg G75 to L21	Sows	Colostrum (U/mL): SOD (119.2 ± 4.14 → 154.8 ± 8.99) ↑ CAT (2.4 ± 0.58 → 18.73 ± 1.1) ↑ GPx (155.3 ± 9.76 → 201.4 ± 18.73) ↑ T-AOC (2.15 ± 0.15 → 2.72 ± 0.12) ↑	[84]
	200 mg/kg G1 to G114	Sows (3 to 5 parity, 226.18 ± 1.09 kg BW on G35 239.64 ± 0.83 kg BW on G85)	Serum (U/mL): G35: GPx (1,169.9 → 1,486.8) ↑ SOD (94.39 → 99.92) ↑; CAT (12.21 → 14.5) ↑ T-AOC (6.03 → 7.12) ↑ G85: SOD (62.28 → 69.58) ↑, T-AOC (5.53 → 7.7) ↑	[85]
Resveratrol	90 mg/kg 21 d	Diquat-challenged weaned piglets 28-day-old 7.25 ± 0.13 kg BW	Jejunal mucosa (U/mg protein): CAT (5.56 → 6.68) ↑, SOD (50.33 → 74.48) ↑ GPx (46.25 → 59.64) ↑ MDA (0.96 → 0.5 nmol/g protein) ↓	[86]
	100 mg/kg 14 d	Diquat-challenged weaned piglets 35-day-old 9.35 ± 0.26 kg BW	Jejunum: T-AOC (0.18 → 0.33 U/mg protein) ↑ H_2_O_2_ (20.43 → 15.61 mmol/g protein) ↓ MDA (1.05 → 0.59 nmol/mg protein) ↓	[87]
	300 mg/kg 28 d	DON-challenged weaned piglets 21-day-old 6.97 ± 0.1 BW	Serum: GSH (2.8 → 3.38 mg/L) ↑ T-AOC (1.56 → 2.01 mg/L) ↑ MDA (3.31 → 2.58 nmol/mL) ↓ Ileum (μmol/g protein): SOD (39.57 → 50.77) ↑, T-AOC (0.31 → 0.44) ↑, MDA (1 → 0.68) ↓ Jejunum (μmol/g protein): GSH (10.02 → 11.17) ↑, SOD (49.95 → 57.76) ↑ T-AOC (0.29 → 0.37) ↑	[36, 37]
		Oxidized soybean oil challenged piglets Average 34.43-day-old 10.19 ± 0.1 kg BW	Plasma H_2_O_2_ (82.99 → 70.11 mmol/L) ↓	[88]
	300 mg/kg G75 to L21	Sows at high temperatures (average parity 3)	Plasma: L14: T-AOC (0.1 → 0.15 mmol/L) ↑ MDA (2.1 → 1.6 nmol/mL) ↓ Colostrum: MDA (3.8 → 2 nmol/mL) ↓	[22]
	300 mg/kg G20 to L21	Sows (average parity 4.4)	Plasma: G110: CAT (12 → 14 U/mL) ↑, GPx (2,200 → 2,800 U/mL) ↑ MDA (2.5 → 1.5 nmol/mL) ↓, H_2_O_2_ (50 → 38 mmol/L) ↓ L14: SOD (80 → 120 U/mL) ↑; CAT (2.5 → 4 U/mL) ↑ MDA (1.6 → 1.2 nmol/mL) ↓, H_2_O_2_ (18 → 15 mmol/L) ↓ L21: SOD (80 → 120 U/mL) ↑; MDA (1.7 → 1.4 nmol/mL) ↓ Placenta (U/mg protein): CAT (28 → 40) ↑, SOD (28 → 48) ↑, GPx (75 → 90) ↑, MDA (5.8 → 3) ↓, H_2_O_2_ (18 → 15 mmol/g protein) ↓	[89]
Pterostilbene	300 mg/kg 15 d	DON-challenged weaned piglets 21-day-old 6.57 ± 0.32 kg BW	Liver: MnSOD (25.5 ± 4.96 → 53.75 ± 5.39 U/mg protein) ↑ GSH (63.11 ± 6.95 → 88.78 ± 6.34 nmol/100 mg wet weight) ↑	[90]
	300 mg/kg 14 d	IUGR piglets 21-day-old 4.91 ± 0.06 kg BW	Jejunum: SOD (60 → 90 U/mg protein) ↑ GSH (1.8 → 2.8 μmol/100 mg wet weight) ↑ T-GSH (3 → 4 μmol/100 mg wet weight) ↑ MDA (0.6 → 0.45 nmol/mg protein) ↓	[91]
Curcumin	400 mg/kg 24 d	IUGR piglets 26-day-old	Serum: T-AOC (1.03 ± 0.37 → 1.89 ± 0.57 U/mL) ↑ CAT (1.77 ± 0.43 → 3.59 ± 1.27 U/mL) ↑ GPx (355.29 ± 50.57 → 376.87 ± 25.39 U/mL) ↑ GR (21.47 ± 1.38 → 30.14 ± 1.24 U/L) ↑ Liver: T-AOC (0.88 ± 0.14 → 1.21 ± 0.22 U/mg protein) ↑ GPx (36.23 ± 2.04 → 58.30 ± 5.17 U/mg protein) ↑ GR (16.11 ± 1.22 → 18.94 ± 3.36 U/g protein) ↑	[92]
	200 mg/kg 89 d	IUGR piglets 26-day-old 5.76–6.05 kg BW	Jejunum: SOD (15.82 ± 3.04 → 28.65 ± 4.83 U/mg protein) ↑ MDA (2.01 ± 0.15 → 1.23 ± 0.13 nmol/mg protein) ↓ Leg muscle (U/mg protein): SOD (16.69 ± 0.23 → 17.83 ± 0.28) ↑ GPx (13.65 ± 0.53 → 15.50 ± 0.82) ↑ CAT (14.18 ± 0.32 → 18.21 ± 0.97) ↑ MDA (4.65 ± 0.32 → 3.42 ± 0.19 nmol/mg protein) ↓	[93, 94]
Polyphenols	Dosages and duration	Swine models	Antioxidant effects	References
Curcumin	200 mg/kg 169 d	IUGR piglets 26-day-old	*longissimus dorsi* muscle GSH (27.99 ± 2.21 → 40.43 ± 1.80) ↑ CAT (10.56 ± 0.58 → 12.59 ± 0.83) ↑ T-AOC (0.32 ± 0.04 → 1.22 ± 0.38) ↑ MDA (2.57 ± 0.17 → 1.08 ± 0.08 nmol/mg protein) ↓	[95]
Eucommia ulmoides flavones	100 mg/kg 21-d feeding	DON-challenged weaned piglets 21-day-old 6.50 ± 0.29 kg BW	Serum (U/mL): D 14: SOD (73.51 ± 6.31 → 94.87 ± 3.37) ↑ GPx (335.21 ± 4.14 → 360.85 ± 2.49) ↑ CAT (5.73 ± 0.38 → 6.97 ± 0.38) ↑ T-AOC (0.54 ± 0.01 → 1.14 ± 0.1) ↑ D 21: GPx (314.49 ± 5.68 → 334.15 ± 4.41) ↑	[96]
	100 mg/kg 14-d feeding		Jejunum: SOD (7.678 ± 2.068 → 13.263 ± 1.67 U/mg protein) ↑ MDA (1.698 ± 0.164 → 3.356 ± 0.546 nmol/mg protein) ↓	[97]

*BW* Body weight, *SOD* Superoxide dismutase, *Gpx* Glutathione peroxidase, *CAT* Catalase, *T-AOC* Total antioxidant capacity, *MDA* Malondialdehyde, *H*_*2*_*O*_*2*_ Hydrogen peroxide, *GSH* Glutathione, *GR* Glutathione reductase

#### Daidzein

Daidzein is commonly used in pigs for its antioxidant properties [98]. Dietary supplementation with 50 mg/kg daidzein improved growth performance, increased SOD activity and decreased plasma MDA in weaning pigs [82]. Dietary supplementation with a high dose of daidzein (640 mg/kg) increased the antioxidant capacity of the longissimus muscle, but had a pro-oxidant effect on the back fat, abdominal fat, liver and plasma of finishing pigs [83]. Moreover, 40 mol/L of daidzein increased the expression of the Nrf2, CAT, and occludin genes in H_2_O_2_-stimulated IPEC-J2 cells [82], suggesting that dietary daidzein may have a beneficial role in the health of the intestine in pigs.

Daidzein is also a type of active phytoestrogen that is beneficial to reproductive performance in sows. In our previous studies, dietary supplementation with 40 mg/kg daidzein from G 75 to L 21 markedly increased the activities of antioxidant enzymes in sow colostrum, while had no effect on serum antioxidant capacity in pregnant sows [84]. Notably, dietary supplementation with 200 mg/kg daidzein may increase serum antioxidant capacity in pregnant sows [85]. We speculated that daidzein is needed more during pregnancy than during lactation to support placenta development.

#### Resveratrol and its derivatives

Resveratrol (3,4',5-trihydroxystilbene) is known for its antioxidant properties in livestock, particularly pigs [99]. For example, dietary resveratrol at 90 mg/kg effectively reduced diquat-induced oxidative stress in piglets through activation of Nrf2 pathways [86]. Resveratrol (15 mol/L) was also found to reduce intracellular ROS accumulation and increase cell viability in IPEC-J2 cells through activation of the Nrf2 pathway [100]. Both in vivo and in vitro experiments show that resveratrol modulates mitophagy in DON-injured piglets [101]. Dietary resveratrol (100 mg/kg) increased intestinal redox status by inducing intestinal mitophagy in diquat-challenged piglets [87]. Furthermore, dietary resveratrol (300 mg/kg) can also ameliorate oxidative stress induced by oxidized soybean oil [88] or DON [36, 37] by altering the intestinal microbiota. These results suggest that different doses of resveratrol may alleviate oxidative stress in weaned piglets through different pathways.

Previous research found that dietary 300 mg/kg resveratrol during pregnancy and lactation increased antioxidant capacity in sows [89]. Similarly, our recent studies found that maternal resveratrol at 300 mg/kg during late gestation and lactation significantly increased sow plasma T-AOC from lactation and decreased sow plasma and colostrum MDA at high summer temperatures, influenced by gut microbiota [22]. However, there is still a lack of studies focusing on the antioxidant effects of other doses of resveratrol in sows, which is in need of further investigation.

Pterostilbene is a methylated derivative of resveratrol, which has recently been used in pig production because of its superior antioxidant effects compared to resveratrol [90, 91, 102]. However, while resveratrol has been under investigation in pig production for almost 10 years [103], pterostilbene has only been under investigation for 3 years [90]. Further studies are needed to investigate the antioxidant effects of pterostilbene and other resveratrol derivatives in pig production.

#### Curcumin

Curcumin, a natural lipophilic polyphenol derived from the turmeric rootstock, has been shown to possess antioxidant properties in swine [104]. Dietary supplementation with 400 mg/kg curcumin increased hepatic antioxidant capacity by increasing the expression of Nrf2 and Hmox1, resulting in improved growth performance in weaned piglets with IUGR [92]. Similarly, dietary curcumin supplementation at 200 mg/kg can improve redox status by activating the Nrf2 signaling pathway in leg muscles, *longissimus dorsi* and jejunum [93–95], which may improve the meat quality or reduce intestinal damage respectively in growing IUGR pigs. Curcumin significantly reduced oxidative stress-induced intestinal damage and mitochondrial dysfunction in piglets by promoting Parkin-dependent mitophagy [105]. Moreover, curcumin reduced diquat-induced intestinal oxidative damage and mitochondrial dysfunction by reducing endoplasmic reticulum stress and inhibiting apoptosis [106]. These results suggest that curcumin alleviates oxidative stress in pigs by ameliorating mitochondrial dysfunction, endoplasmic reticulum stress and apoptosis, and by activating the Nrf2 signaling pathway. The recommended dose of curcumin for pigs is (200 to 400 mg/kg body weight).

#### *Eucommia ulmoides* flavones

*Eucommia ulmoides *flavones (EUFs) are secondary metabolites from *Eucommia ulmoides* (a Chinese herb with various medicinal properties [107]) which have recently been shown to have potent antioxidant activities in pigs. Dietary supplementation with 100 mg/kg EUF attenuated diquat-induced oxidative stress, inflammatory response and impaired growth in piglets [96]. The Nrf2 signaling pathway has been shown to play a critical role in EUF-mediated alleviation of intestinal oxidative stress in diquat-treated piglets. [97]. These findings may have implications for the investigation of EUFs as potential antioxidants in pig production. However, further studies are needed to investigate whether and how EUFs exert antioxidant effects in pigs at other stages of life, such as in finishing pigs and in perinatal sows.

Taken together, dietary supplementation with polyphenols may have good antioxidant effects in pigs. However, the underlying mechanism by which polyphenols exert antioxidant effects is in need of further investigation.

### Functional amino acids

**Fig. 3:**
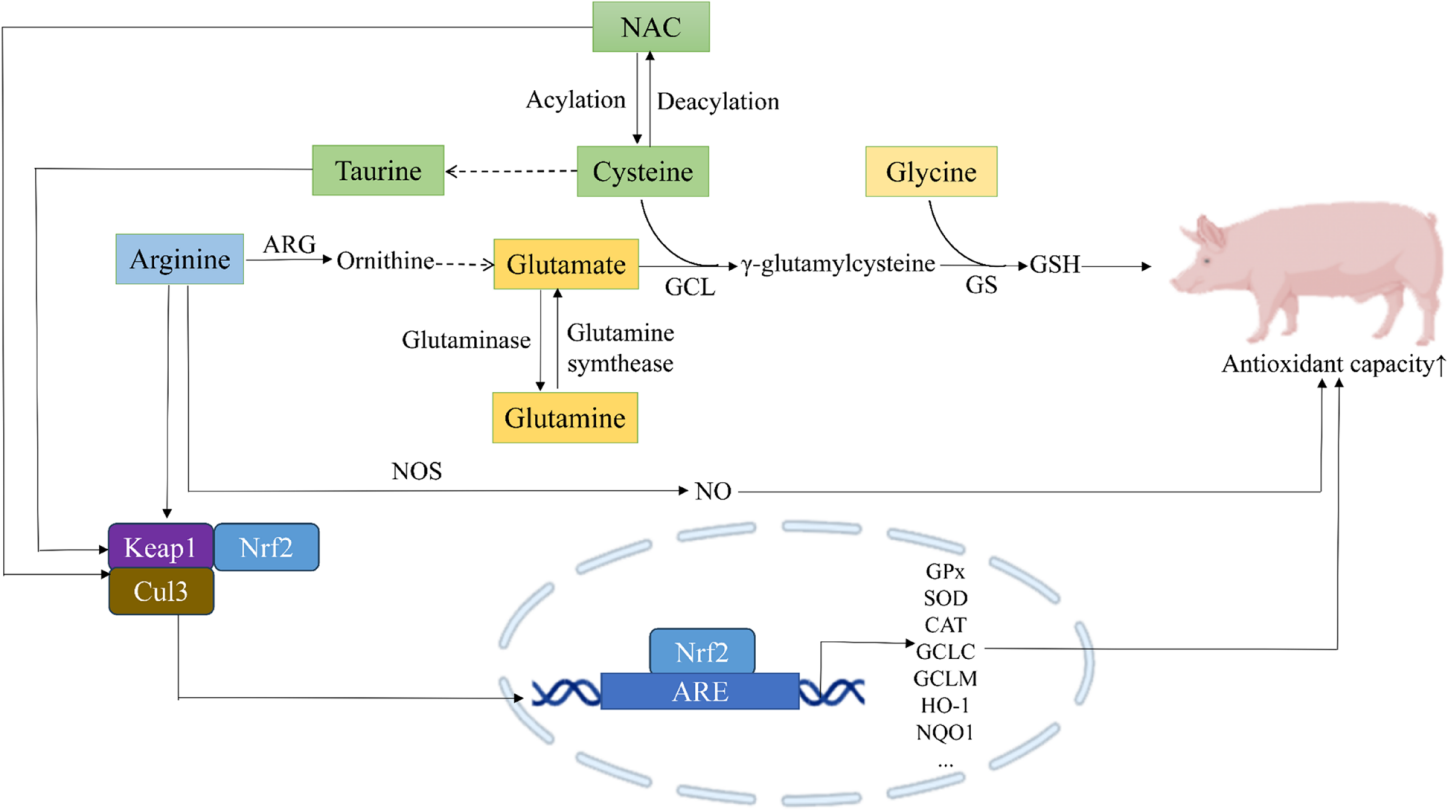
The proposed molecular mechanisms by which functional amino acids improve the antioxidant capacity of swine. Arginine supplementation in swine can be catalyzed to ornithine, and then transfer into glutamate, which conjugates cysteine to generate γ-glutamyl cysteine that finally conjugates glycine to form GSH to improve the antioxidant capacity. Supplemented with glutamate, cysteine and glycine, or its precursors glutamine and NAC in swine can also improve the antioxidant capacity via GSH synthesis. Arginine, taurine and NAC can perform antioxidant effects through the activation of Nrf2 pathway, and arginine can also improve antioxidant capacity of swine via NO synthesis stimulated by NOS. NAC: *N*-acetylcysteine; ARG: arginase; NOS: oxide synthase; NO: nitric oxide; GS: synthase; GSH: glutathione; Nrf2: nuclear factor erythroid 2 (NF-E2)-related factor 2; Keap1: Kelch-like ECH-associated protein 1; Cul3: Cullin3; ARE: antioxidant responsive element; GPx: glutathione peroxidase; SOD: superoxide dismutase; CAT: catalase; GCLC: glutamate cysteine ligase catalytic; GCLM: glutamate cysteine ligase modifier; HO-1: heme oxygenase; NQO1: NAD(P)H:quinone oxidoreductase 1

Functional amino acids and their bioactive precursors such as nitric oxide (NO), polyamines and GSH could reduce oxidative stress and improve pig growth, reproduction and health (Fig. 3) [108, 109]. For example, supplementation with 1% arginine dramatically increased antioxidant capacity in serum and skeletal muscle to improve meat quality in growing pigs [110]. Dietary supplementation with 0.8% and 1.6% arginine significantly increased plasma and liver GPx and SOD activities in diquat-challenged weaned piglets to alleviate oxidative stress responses [111]. It is interesting to note that arginine could be metabolized to NO to alleviate oxidative stress in sows [112–114]. Furthermore, our previous studies showed that arginine administration significantly increased GPx activity and reduced ROS and MDA production via the arginase-1 pathway in LPS-stimulated IPEC-J2 cells [115].

Cysteine, glutamate and glycine are precursors of GSH synthesis. Therefore, these three amino acids may influence antioxidant function [116]. Cysteine supplementation increased intestinal CAT, SOD, GPx and GSH activities for maintenance of intestinal integrity in LPS-challenged weaned piglets [117]. Supplementation with 1.2 g/kg *N*-acetylcysteine (NAC) (a precursor of cysteine) increased GSH levels and decreased MDA levels in the liver of IUGR suckling piglets [118]. Dietary supplementation with 500 mg/kg NAC increased liver antioxidant capacity in weanling piglets to attenuate LPS-induced liver injury [61]. Treatment with 800 μmol/L NAC increased the antioxidant capacity to reduce cell apoptosis in H_2_O_2_-induced IPEC-J2 cells [68], suggesting that NAC could rescue H_2_O_2_-induced intestinal oxidative damage in piglets. Moreover, treatment with 5 mmol/L NAC attenuated 4-HNE-induced cell death in intestinal epithelial cells through activating the Nrf2 signaling pathway [53]. Furthermore, increased supplementation of cysteine in the diet (from 0.3% to 0.4% and 0.5%) could reduce plasma MDA levels to reduce oxidative stress in sows during late gestation and lactation [119]. Dietary NAC supplementation (500 mg/kg) significantly increased serum and placental antioxidant capacity (e.g., GSH levels) and activated the STAT3/occludin/ZO-1 pathway in the placentas of sows to attenuate DON-induced placental oxidative stress and barrier damage, further reducing the incidence of stillbirths and low birth weight piglets [120].

Glutamate also possesses antioxidant properties [121]. Dietary supplementation with 2% glutamate increased plasma SOD and GPx activity and promoted intestinal epithelial cell proliferation in DON-challenged piglets [122]. Furthermore, glutamate supplementation reduced MDA production and ameliorated diquat-induced oxidative stress in piglets by increasing SOD, T-AOC and NO levels while decreasing MDA production [57]. Furthermore, dietary supplementation with glutamine (a precursor of glutamate) can increase intestinal antioxidant capacity [123] and the expression of genes involved in GSH synthesis (e.g., glutamate cysteine ligase catalytic (*GCLC*), glutamate cysteine ligase modifier (*GCLM*), and glutathione reductase (*GSR*)) and 4-HNE metabolism (e.g., glutathione S-transferase A 1 (*GSTA1*) and *GSTA4*) to attenuate 4-HNE-induced oxidative stress [55].

Glycine, one of the key components of GSH, is of great importance in the treatment of oxidative stress in pigs. Dietary 0.5%–2% glycine supplementation linearly increased plasma and intestinal glycine and GSH levels in milk-fed piglets [124]. Dietary supplementation with 1% glycine improved intestinal mucosal morphology and antioxidant capacity (e.g., GSH and GPx activities), and inhibited the ferroptosis through regulating key proteins (e.g., transferrin receptor 1, SLC7A11 and GPx4) in intestinal mucosa of diquat-challenged weanling piglets [125]. Furthermore, treatment with 1 mmol/L glycine significantly increased GSH activity, protein synthesis and cell proliferation, and decreased cell apoptosis in the 4-HNE-induced oxidative stress model of IPEC-1 cells [126]. These results suggest that glycine may inhibit lipid peroxidation and intestinal epithelial ferroptosis by reducing oxidative stress in piglets through the production of GSH and GPx.

Taurine is an amino acid derived from converting sulphur-containing amino acids. Dietary taurine levels of 0.3% to 0.6% increased the activity of antioxidant enzymes (e.g., T-SOD and GPx) and reduced the levels of oxidative indicators (ROS, 8-OHdG and MDA) by increasing and activating the Nrf2 signaling system in weaned piglets [127]. Besides, dietary supplementation with 1% taurine from G 75 to lactation significantly increased the antioxidant capacity in the gilts' milk and the intestinal antioxidant effects in the piglets [128]. Furthermore, pre-treatment with 2 mmol/L taurine reduced H_2_O_2_-induced oxidative stress in PMECs by increasing SOD activity and decreasing intracellular ROS [129]. These results suggest that dietary taurine supplementation may improve antioxidant capacity and milk performance via the Nrf2 pathway in lactating sows.

In conclusion, dietary supplementation with functional amino acids has potential antioxidant effects in pigs, but this requires further investigation using different models of oxidative stress, functional amino acids, and dietary levels or proportions of functional amino acids.

## Conclusions and perspectives

In this review, we focused on research into the dose and dose intensity of the causes, models and nutritional strategies for oxidative stress in pigs. However, there are still many pressing issues that need to be addressed in the field of oxidative stress models and nutritional regulation in pigs: (1) Ideal levels of ROS are essential for maintaining animal health, so we should first find the ranges of ROS levels in pigs that are most beneficial to their health before determining the appropriate amount of antioxidants in the diet; (2) At present, the main method used to determine whether pigs are under oxidative stress is to compare the relevant indexes of oxidative stress in the control group. In the future, existing data and new approaches should be used to determine the reference range of oxidative indexes; (3) A systemic comparison of the effects of different causes of oxidative stress should be carried out in the future; and (4) Nutritional strategies for oxidative stress in pigs should be thoroughly investigated. It is important to note that oxidative stress can reduce feed intake in pigs and it is unclear whether increasing nutrient density or changing the proportion of nutrients in the diet can eliminate the negative effects of oxidative stress. We hope that this review will serve as a theoretical basis and reference for the development of accurate models and nutritional strategies for specific oxidative stress.

## Data Availability

Not applicable.

## Abbreviation

AFB1: Aflatoxin B1
BW: Body weight
CAT: Catalase
CTX: Cyclophosphamide
DON: Deoxynivalenol
EUFs: *Eucommia ulmoides* flavones
G: Gestation
GCLC: Glutamate cysteine ligase catalytic
GCLM: Glutamate cysteine ligase modifier
GPx: Glutathione peroxidase
GSH: Glutathione
GSR: Glutathione reductase
GSSG: Glutathione oxidized
GSTA: Glutathione S-transferase A
4-HNE: 4-Hydroxynonenal
HO-1: Heme oxygenase 1
H_2_O_2_: Hydrogen peroxide
IPEC: Intestinal porcine epithelial cell line
IUGR: Intrauterine growth restriction
L: Lactation
LPS: Lipopolysaccharide
MDA: Malondialdehyde
MLNs: Mesenteric lymph nodes
NAC: *N*-Acetylcysteine
NF-κB: Nuclear factor-κB
NO: Nitric oxide
NQO1: NAD(P)H:quinone oxidoreductase 1
Nrf2: Nuclear factor erythroid 2-related factor 2
8-OHG: 8-Hydroxyguanosine
8-OHdG: 8-Hydroxy-2 deoxyguanosine
OTA: Ochratoxin A
PMECs: Porcine mammary epithelial cells
RLU: Relative light unit
ROS: Reactive oxygen species
SOD: Superoxide dismutase
STAT: Signal transducer and activator of transcription
T-AOC: Total antioxidant capacity
TBARS: Thiobarbituric acid reactive substances
UCP2: Uncoupling protein 2
ZO-1: Zonula occludens-1
